# Rice Straw-Derived Biochar Mitigates Microcystin-LR-Induced Hepatic Histopathological Injury and Oxidative Damage in Male Zebrafish via the Nrf2 Signaling Pathway

**DOI:** 10.3390/toxins16120549

**Published:** 2024-12-18

**Authors:** Wang Lin, Fen Hu, Wansheng Zou, Suqin Wang, Pengling Shi, Li Li, Jifeng Yang, Pinhong Yang

**Affiliations:** 1 College of Life and Environmental Sciences, Hunan University of Arts and Science, Changde 415000, China; linwang@huas.edu.cn (W.L.); hufen1206@163.com (F.H.); zwsksy@126.com (W.Z.); suqinwanghn@outlook.com (S.W.); shuichan051@126.com (P.S.); 2 Institute for Ecological Research and Pollution Control of Plateau Lakes, School of Ecology and Environmental Science, Yunnan University, Kunming 650500, China; 3 Hunan Provincial Key Laboratory for Molecular Immunity Technology of Aquatic Animal Diseases, Changde 415000, China; 4 College of Fisheries, Huazhong Agricultural University, Wuhan 430070, China; foreverlili78@mail.hzau.edu.cn; 5 College of Chemistry and Materials Engineering, Hunan University of Arts and Science, Changde 415000, China

**Keywords:** microcystin-LR, rice straw-derived biochar, histopathological analysis, oxidative damage, zebrafish

## Abstract

Microcystin-leucine arginine (MC-LR) poses a serious threat to aquatic animals during cyanobacterial blooms. Recently, biochar (BC), derived from rice straw, has emerged as a potent adsorbent for eliminating hazardous contaminants from water. To assess the joint hepatotoxic effects of environmentally relevant concentrations of MC-LR and BC on fish, male adult zebrafish (*Danio rerio*) were sub-chronically co-exposed to varying concentrations of MC-LR (0, 1, 5, and 25 μg/L) and BC (0 and 100 μg/L) in a fully factorial experiment. After 30 days exposure, our findings suggested that the existence of BC significantly decreased MC-LR bioavailability in liver. Furthermore, histopathological analysis revealed that BC mitigated MC-LR-induced hepatic lesions, which were characterized by mild damage, such as vacuolization, pyknotic nuclei, and swollen mitochondria. Compared to the groups exposed solely to MC-LR, decreased malondialdehyde (MDA) and increased catalase (CAT) and superoxide dismutase (SOD) were noticed in the mixture groups. Concurrently, significant changes in the mRNA expression levels of Nrf2 pathway genes (*cat*, *sod1*, *gstr*, *keap1a*, *nrf2a*, and *gclc*) further proved that BC reduces the oxidative damage induced by MC-LR. These findings demonstrate that BC decreases MC-LR bioavailability in the liver, thereby alleviating MC-LR-induced hepatotoxicity through the Nrf2 signaling pathway in zebrafish. Our results also imply that BC could serve as a potentially environmentally friendly material for mitigating the detrimental effects of MC-LR on fish.

## 1. Introduction

The severity of toxic algae blooms in surface waters has intensified due to eutrophication and global warming in recent decades [1]. Cyanobacterial blooms typically cause foul odors and dissolved oxygen deficits in water bodies [2]. Additionally, secondary metabolites, such as microcystins (MCs), are released during toxic cyanobacterial cell decay and collapse, adversely affecting aquatic ecosystems [3]. More than 300 MCs congeners with varying structures have been discovered [4], with microcystin-LR (MC-LR) being the most concerning. MC-LR is highly stable and resistant to natural purification processes due to its cyclic structures and Adda side-chain [5]. Dissolved MC concentrations have been reported to reach levels of 17 μg/L MC-LR equivalents in Taihu Lake [6]. In Ain Zada reservoir, Algeria, MC-LR concentrations surge to 19.6 μg/L during cyanobacteria blooms, greatly exceeding the provisional guideline value of MC-LR (1.0 μg/L) established by WHO [7]. Currently, the probability of exposure to MC-LR for both aquatic animals and humans is significant in daily life [8,9], highlighting the importance of understanding its detrimental effects at environmentally relevant concentrations. Numerous studies have demonstrated that MCs can accumulate in fish tissues, leading to a range of toxic effects, including hepatotoxicity, developmental toxicity, and reproductive toxicity [10,11,12,13,14,15]. These adverse effects not only threaten fish health but also pose risks to public health through the consumption of contaminated fish.

The liver is known as the principal target organ of MC-LR, where MC-LR mainly accumulates [16]. The hepatotoxicity of MC-LR is mainly caused by the inhibition of serine/threonine protein phosphate 1 and 2A [17]. Furthermore, Shi et al. [18] found that MC-LR can induce the excessive production of reactive oxygen species (ROS) and oxidative stress, thereby contributing to hepatotoxicity. Both acute and chronic MC-LR exposure can result in oxidative injury, mainly characterized by an oxidant–antioxidant imbalance [19]. To date, a growing number of studies have focused on investigating the enhanced or even synergistic toxicity to aquatic organisms when they are co-exposed to MC-LR in combination with various contaminants, such as inorganic nitrogenous pollutants [20], persistent organic pollutants [21], and microplastics [22]. Nevertheless, few researchers have evaluated the joint toxicity of MC-LR and other adsorbent materials, which have the potential to treat water pollution, in fish.

Biochar (BC) is a porous material with high adsorption properties, produced by pyrolyzing biomass under oxygen-limiting conditions [23]. In addition to its adsorption capabilities, BC can interact with adsorbates through electrostatic attraction and hydrogen bonding [24]. BC is widely recognized for effectively adsorbing and removing organic and inorganic contaminants from wastewater [25]. It was reported that BC derived from chicken manure, which has a higher ash content, is highly effective at adsorbing and removing aqueous MC-LR [26]. Moreover, BC produced from rice straw also exhibits high adsorption capacity for MC-LR removal, with capacity increasing as the pyrolysis temperature rises [27]. Due to its high adsorption capacity and wide availability, BC is increasingly used to remove pollutants from aquatic environments and has the potential to mitigate MC-LR contamination during cyanobacterial blooms. However, the potential impact of BC and its adsorbates on fish remains underexplored and needs further elucidation.

Zebrafish are a well-established model organism frequently used in toxicological research on aquatic pollutants [28]. In this study, we conducted a 30-day co-exposure experiment to investigate the potential mitigating effects of BC on MC-LR-induced hepatotoxicity in adult male zebrafish. Our primary objectives were to evaluate hepatic pathological lesions and alterations in antioxidant parameters, as well as to explore the underlying molecular mechanisms related to antioxidant function in zebrafish co-exposed to MC-LR and BC. The results of this study will provide novel insights into the practicality of utilizing BC for the removal of MC-LR during cyanobacterial blooms, which is of great significance for water environment restoration and human health.

## 2. Results

### 2.1. Characterization of BC

**Figure 1:**
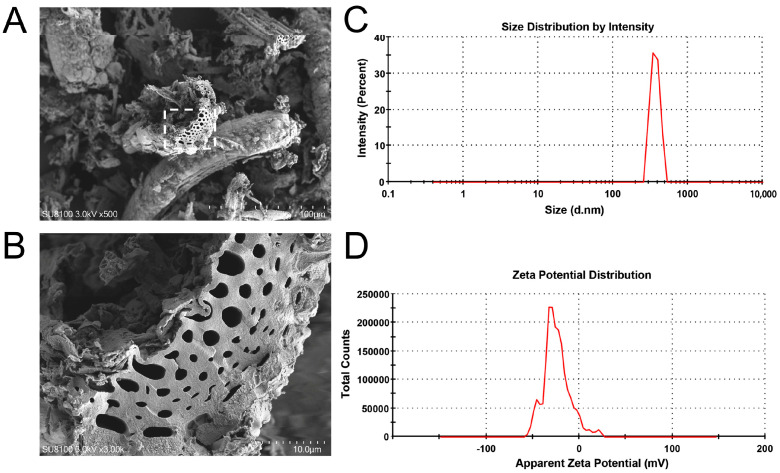
SEM images (**A**,**B**), size distribution (**C**), and zeta potential (**D**) of BC.

SEM images of rice straw-derived BC are shown in Figure 1A,B, where the irregular porous structures are visually evident. Moreover, as shown in Figure 1C,D, the measured average diameter of BC was 847.6 ± 50.28 nm, and the zeta potential of BC was −25.2 ± 20.8 mV.

### 2.2. MC-LR Levels in Water and Liver Tissues

As shown in Table S1, SI, compared to the MC-LR-only groups, significant decreases in MC-LR levels in water samples before water renewal were observed in the co-exposure groups (*p* < 0.01). Furthermore, compared to the 25 μg/L MC-LR group, the MC-LR contents that accumulated in the livers also exhibited a significant reduction in the mixture group (*p* < 0.01) (Table S1, SI).

### 2.3. Histopathological Observations

**Figure 2:**
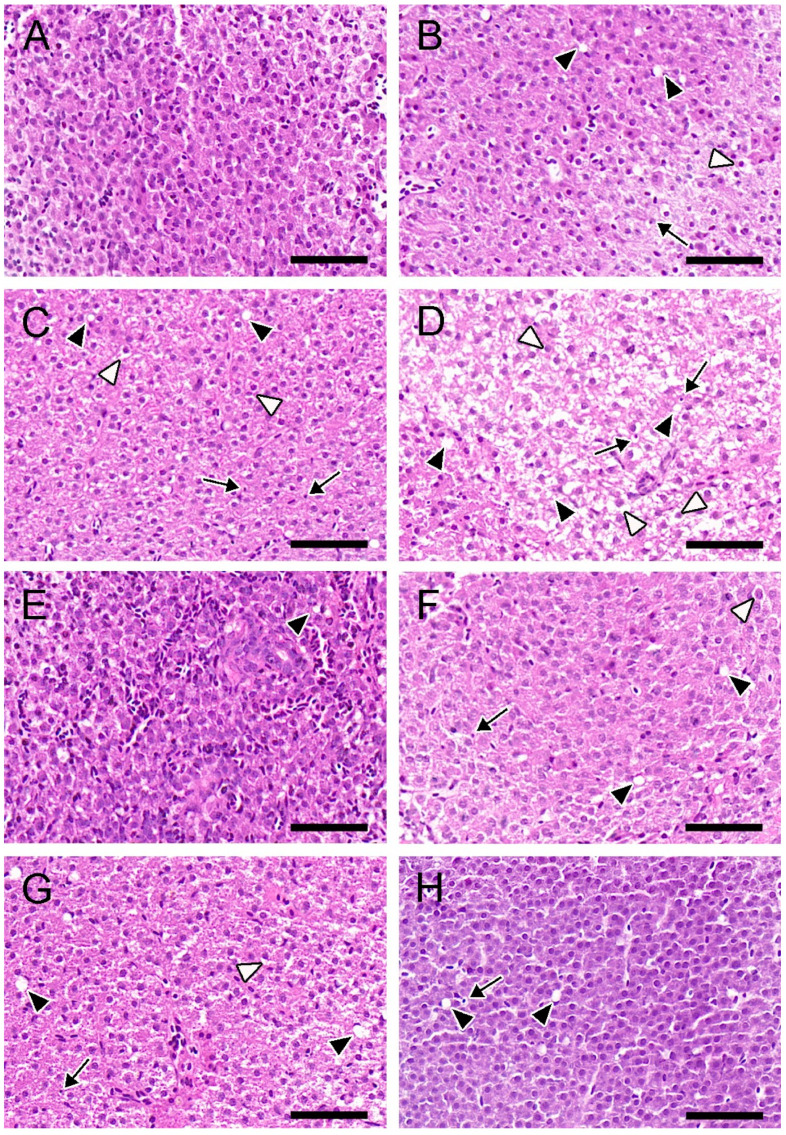
Hepatic H&E-stained sections of zebrafish. Control (**A**); 1 μg/L MC-LR (**B**); 5 μg/L MC-LR (**C**); 25 μg/L MC-LR (**D**); 100 μg/L BC (**E**); 1 μg/L MC-LR+ BC (**F**); 5 μg/L MC-LR + BC (**G**); and 25 μg/L MC-LR + BC (**H**). Pyknotic nuclei (black arrow), swollen hepatocytes (white arrowhead), and vacuolization (black arrowhead). Bar = 50 um.

As shown in Figure 2A, the livers in the control group displayed dense cytoplasm and clear nuclei. In contrast, hepatic lesions were observed and aggravated with increasing MC-LR concentrations, which were characterized by cell vacuolization, pyknotic nuclei, and swollen hepatocytes (Figure 2B–D). In the BC-only group, the liver tissues exhibited a well-organized structure, with only slight vacuolization observed (Figure 2E). In the mixture groups, the hepatic histopathological injury was reduced, primarily characterized by a lower occurrence of vacuolization and relatively mild swollen hepatocytes and pyknotic nuclei (Figure 2F–H). Compared to the 25 μg/L MC-LR-only group, the semi-quantitative analysis of hepatic H&E-stained sections showed that the degree of hepatic injury was alleviated in the 25 μg/L MC-LR + BC group, which characterized only by vacuolization (++) and pyknotic nuclei (+) (Table 1).

**Figure 3:**
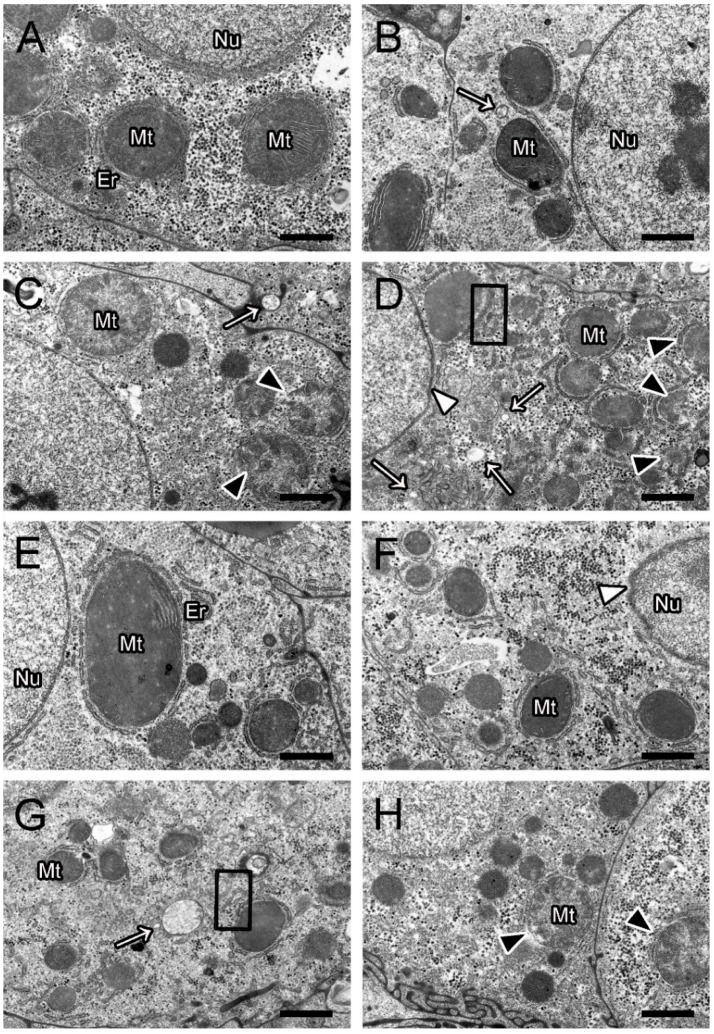
Hepatic ultrastructural alterations in zebrafish. Control (**A**); 1 μg/L MC-LR (**B**); 5 μg/L MC-LR (**C**); 25 μg/L MC-LR (**D**); 100 μg/L BC (**E**); 1 μg/L MC-LR + BC (**F**); 5 μg/L MC-LR+ BC (**G**); and 25 μg/L MC-LR + BC (**H**). Swollen mitochondria (black arrowhead); deformed nuclei (white arrowhead); vacuolization (white arrow); and dilated endoplasmic reticulum (black box). Mt, mitochondria; Nu, nuclei; ER, endoplasmic reticulum. Bar = 1 μm.

Hepatic ultrastructural pathological observation in the control group exhibited a normal structure, with round nuclei and intact mitochondria containing dense matrices (Figure 3A). Furthermore, no significant lesions were found in the BC-only group (Figure 3E). Hepatic injuries, including swollen mitochondria, vacuolization, deformed nuclei, and dilated endoplasmic reticulum, were observed and became more severe with increasing MC-LR concentrations (Figure 3B–D). In the mixture groups, the TEM images illustrated that the MC-LR-induced hepatic ultrastructural lesions were alleviated in the presence of BC (Figure 3F–H). Compared to the 25 μg/L MC-LR-only group, the semi-quantitative analysis also confirmed that hepatic ultrastructural damage was greatly alleviated and only characterized by swollen mitochondria (++) in the corresponding mixture group (Table 2).

### 2.4. Antioxidant Parameter Analysis

**Figure 4:**
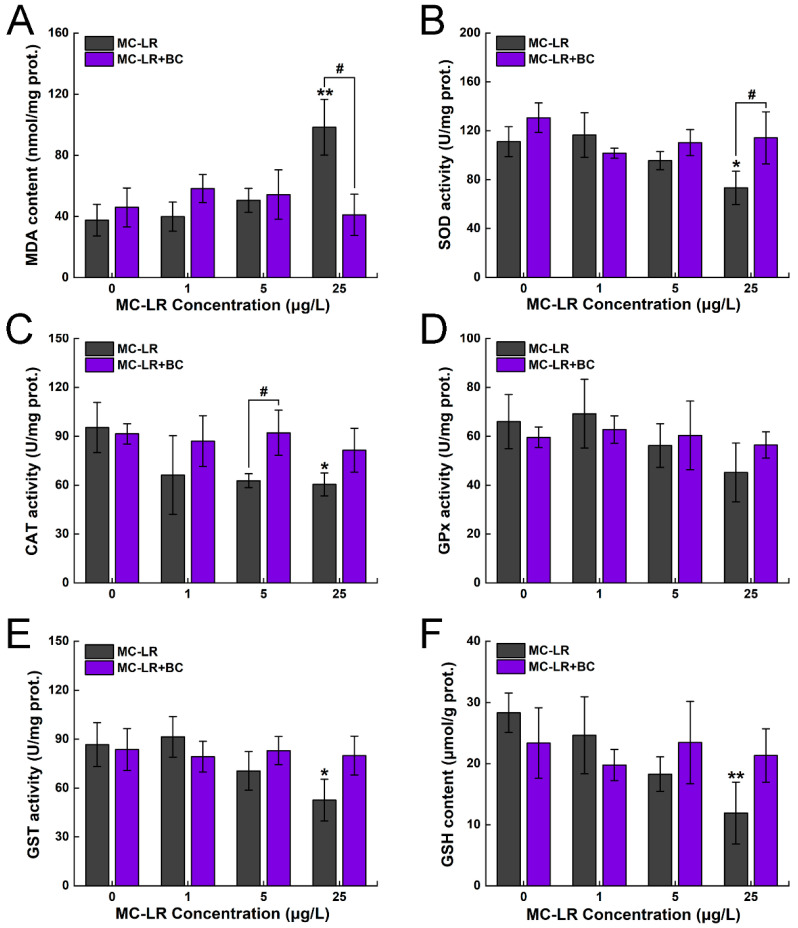
Changes in MDA content (**A**), SOD activity (**B**), CAT activity (**C**), GPx activity (**D**), GST activity (**E**), and GSH content (**F**) in the liver of zebrafish. Asterisks (* *p* < 0.05; ** *p* < 0.01) indicate significant differences between the treatments and control. Hashes (# *p* < 0.05) indicate significant differences between the MC-LR + BC groups and the MC-LR-only groups, respectively. The values are presented as mean ± SD (n = 3).

In the 25 μg/L MC-LR group, the MDA levels showed a significant increase, with a maximum induction of 162% compared to the control group (*p* < 0.01). However, compared to the 25 μg/L MC-LR group, the MDA levels showed a significant decrease, with a 58% reduction in the corresponding mixture group (*p* < 0.05) (Figure 4A). The activities of SOD and CAT also showed a significant decrease when exposed to 25 μg/L MC-LR (*p* < 0.05) (Figure 4B,C). Moreover, a significant increase in SOD activity was observed in the mixture group compared to the 25 μg/L MC-LR group (*p* < 0.05). Compared to the 5 μg/L MC-LR group, CAT activity exhibited a significant increase in the corresponding combined group (*p* < 0.05). GPx activity showed no significant alterations when exposed to MC-LR only or its combination with BC (Figure 4D). Meanwhile, GST activity exhibited a significant reduction in the 25 μg/L MC-LR group (*p* < 0.05) compared to the control group (Figure 4E). Similarly, GSH showed a significant decrease in the 25 μg/L MC-LR group, with a maximum reduction of 58% (*p* < 0.01) (Figure 4F).

### 2.5. Gene Expression

**Figure 5:**
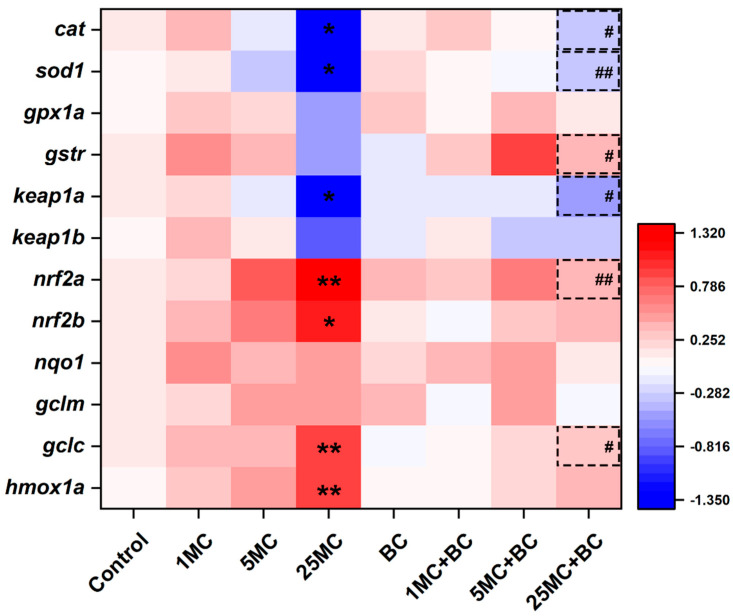
Heatmap of Nrf2 signaling pathway gene expression. Asterisks (* *p* < 0.05; ** *p* < 0.01) indicate significant differences between the treatments and control. The dotted box with hashes (# *p* < 0.05; ## *p* < 0.01) indicate significant differences between the MC-LR + BC groups and the MC-LR-only groups, respectively. The values are presented as mean ± SD (n = 6).

To evaluate the oxidative stress induced by MC-LR and BC, the transcription levels of Nrf2 signaling pathway genes were measured (Figure 5 and Table S2, SI). Compared to the control group, the mRNA levels of *nrf2a* (*p* < 0.01), *nrf2b* (*p* < 0.05), *gclc* (*p* < 0.01), and *hmox1a* (*p* < 0.01) exhibited a significant increase in the 25 μg/L MC-LR group. On the contrary, the transcription levels of *cat*, *sod1*, and *keap1a* were significantly downregulated in the 25 μg/L MC-LR group in comparison to the control group (*p* < 0.05). In the BC-only group, no significant alterations in all tested genes were observed. Compared to the 25 μg/L MC-LR group, *cat*, *gstr*, and *keap1a* were significantly increased (*p* < 0.05), whereas *gclc* was significantly decreased (*p* < 0.05) in the co-exposure group. Moreover, a significant upregulation of *sod1* and a significant downregulation of *nrf2a* were observed in the 25 μg/L MC-LR + BC group compared to the 25 μg/L MC-LR group (*p* < 0.01). No significant alterations in *gpx1a*, *keap1b*, *nqo1*, and *gclm* were observed across all groups.

## 3. Discussion

The detrimental impacts of MC-LR on fish have been widely investigated and have raised public concern. However, the underlying risks of combining MC-LR with BC, a potential adsorbent for removing contaminants, remains poorly understood. The present study provides new insights into the potential use of BC for treating cyanotoxins in natural waters, emphasizing the principles of environmental friendliness and biological safety.

### 3.1. Adsorption Capacity of BC for MC-LR

BC is characterized by several structural properties, including surface functional groups, porous structures, and high specific surface areas [29]. These features enhance its efficiency in adsorbing and removing waterborne contaminants, including inorganic and organic pollutants [25]. Furthermore, different MC congeners exhibit varying polarities, which can affect their adsorption capacity onto adsorbents, ultimately resulting in differing toxicity levels [30]. In our study, SEM images showed that BC has a porous structure. Moreover, BC reduced MC-LR concentrations in the water and its bioavailability in the liver, demonstrating its ability to adsorb and immobilize MC-LR. Wei and Lu [27] suggested that rice straw-derived BC, when pyrolyzed at higher temperatures, exhibits greater adsorption capacity and removal efficiency for MC-LR due to its larger and denser pore structure. Additionally, Zeng and Kan [31] suggested that BC activated with iron (FeCl_3_) can effectively adsorb MC-LR from lake water, reducing its dissolved levels and thereby lowering the potential risk of MC-LR toxicity to aquatic organisms. Thus, eco-friendly BC has proven effective in immobilizing MC-LR and reducing its bioconcentration, potentially alleviating MC-LR-induced toxicity in fish.

### 3.2. Effects of MC-LR and BC on Hepatic Injury

Histopathological lesions in fish are widely used as effective indicators for evaluating the toxicity of aquatic contaminants. The liver, being the principal detoxifying organ, often shows toxic effects at both the tissue and cellular levels under pollutant exposure [32]. In our study, histopathological lesions, including vacuolization and pyknotic nuclei, were observed in the liver when exposed only to MC-LR. Similarly, the acute intraperitoneal (IP) injection of 500 μg/kg MC-LR leads to severe hepatocellular vacuolization and necrosis in tilapia fish (*Oreochromis* sp.) [33]. Additionally, sub-chronic exposure to 10–30 μg/L MC-LR also results in hepatic cytoplasmic vacuolization in zebrafish [34]. The appearance of hepatocyte cytoplasmic vacuolization may represent a degenerative change at the subcellular level, potentially leading to abnormal biological function [35].

Mitochondria are crucial for maintaining cellular redox homeostasis, and they are particularly vulnerable to damage from free radicals [36]. In the present study, mitochondrial swelling and endoplasmic reticulum dilation were observed in the MC-LR-only groups. Mitochondria swelling can lead to enhanced substrate oxidation and elevated ROS production, whereas endoplasmic reticulum dilation indicates endoplasmic reticulum stress, which could eventually result in excessive oxidative stress and apoptosis [37]. Consistent with our findings, Hou et al. [38] also demonstrated that the IP injection of MC-LR causes severe hepatic damage, including vacuolar degeneration, cellular swelling, and results in antioxidant dysfunction in zebrafish. In our study, milder histopathological lesions were observed in the mixture groups, suggesting that the presence of BC alleviates MC-LR-induced hepatic injury. Similarly, histopathological damage to rat liver in the carbofuran-co-BC group was significantly mitigated compared to the single carbofuran group, indicating BC’s effectiveness in adsorbing deleterious pollutants and reducing their toxicity [39]. Thus, our results demonstrate that MC-LR can cause serious tissue damage, whereas the presence of BC can alleviate hepatic injury and help to restore normal function.

### 3.3. Effects of MC-LR and BC on Hepatic Antioxidant Function

The overproduction of MDA is tightly associated with oxidative stress, which is a crucial indicator for assessing hepatic damage [40]. Former research has shown that MC-LR can significantly increase hepatic MDA levels in fish through various exposure routes, such as IP injection and immersion [34,41]. In our study, MDA levels exhibited notable elevation under MC-LR stress but returned to baseline in the co-treatment group, demonstrating that MC-LR induces hepatic oxidative stress, whereas the presence of BC helps to alleviate this damage. Similar to our results, olive stone-derived BC has been shown to remove inorganic mercury (Hg) from water and reduce oxidative damage by lowering MDA levels in Nile tilapia (*Oreochromis niloticus*) [42].

Fish have developed an endogenous antioxidant defense system to counteract oxidative damage, which primarily comprises antioxidant enzymes that neutralize excess oxidants [43]. The activity of these enzymes provides insight into the antioxidant status and overall health of fish under various stress conditions. SOD and CAT are crucial components of the enzymatic defense system, with SOD converting superoxide anions into H_2_O_2_, which is subsequently decomposed into water by CAT [44]. A significant reduction in hepatic SOD and CAT activity in the 25 μg/L MC-LR group was observed in our study, with a similar trend in the mRNA levels of *cat* and *sod1*. Similarly, Ming et al. [45] found a significant reduction in hepatic CAT and SOD in grass carp (*Ctenopharyngodon idella*) under acute MC-LR stress, implying that MC-LR exposure inhibits fish antioxidant function. In the co-exposure groups, both the activities and transcription levels of SOD and CAT returned to normal levels, suggesting that BC mitigated the oxidative damage induced by MC-LR and facilitated the restoration of antioxidant capacity.

The NF-E2-related factor 2/Kelch-like ECH-associated protein 1 (Nrf2/Keap1a) signaling pathway is crucial for activating downstream antioxidant genes to exert antioxidant function in fish and may serve as a potential target for regulating MC-LR hepatotoxicity [46,47]. Nrf2 can activate antioxidant gene expression to help mitigate damage under oxidative stress [48]. Keap1a acts as an intracellular sensor of oxidants and can suppress the hyperactivation of Nrf2 [49]. In our study, the mRNA levels of *nrf2a*, *nrf2b*, *gclc*, and *hmox1a* significantly increased, whereas *keap1a* significantly decreased under MC-LR stress, indicating activation of the protective antioxidant response. Consistent with previous studies, MC-LR exposure was found to activate the Nrf2 signaling pathway as an adaptive mechanism against oxidative damage [50]. Nevertheless, in the mixture groups, a significant increase in *keap1a* and reductions in *nrf2a* and *gclc* were observed relative to the corresponding MC-LR group. Our findings confirm that sub-chronic exposure to MC-LR leads to antioxidant dysfunction through the Nrf2 pathway, whereas BC mitigates the oxidative damage and aids in restoring normal antioxidant function.

In our study, a significant reduction in GSH contents and GST activity in the MC-LR-only group were observed, implying that the GSH pathway was insufficient to scavenge the excessive ROS and exert effective detoxification [51]. Nevertheless, both GST activity and GSH content returned to normal levels in the co-treatment group, further demonstrating that BC mitigated MC-LR-induced GSH depletion. Kumari et al. [52] also found that rice straw-derived BC can restore antioxidant capacity by increasing GSH content in earthworm (*Eisenia fetida*) under chlorpyrifos stress, further confirming that BC can help to alleviate oxidative damage caused by pesticides. In the present study, no significant changes in the antioxidant parameters were noticed in the single BC group, suggesting that 100 μg/L rice straw-derived BC did not affect hepatic antioxidant function. Therefore, considering the eco-friendly and cost-effective nature of BC, our results provide an effective approach to reducing the harm of cyanotoxins for aquatic organisms.

## 4. Materials and Methods

### 4.1. Toxin

MC-LR (purity ≥ 95%) was obtained from Algal Science (Taiwan, China) and was dissolved in ultrapure water to prepare the MC-LR stock solution (0.5 mg/mL).

### 4.2. Characterization of BC

Rice straw was acquired from local farmland (Changde, China) and utilized as raw material in our study. Next, samples were rinsed thoroughly with ultrapure water and dried for 24 h. The dry straws were ground and then pyrolyzed in a muffle furnace (Haoyue Technology, Shanghai, China) at a heating rate of 15 °C/min under oxygen-limited conditions at 400 °C for 3 h. Then, the samples were sieved through a 200-mesh sieve to acquire the final BC. The size distribution and zeta-potential of the rice straw-derived BC were measured using a Zetasizer Nano-ZS (Malvern Instruments, Malvern, UK). Scanning electron microscopy (SEM) (Hitachi, Tokyo, Japan) was used to characterize the morphological structure of BC.

### 4.3. Experimental Design and Sampling

Adult male zebrafish (AB, 3-month-old) were acquired from Zebrafish Resource Center (Wuhan, China). The fish were cultured in tanks and fed fresh *Artemia* nauplii twice daily. The photoperiod was adjusted to 14 h:10 h (light/dark), with the water temperature maintained at 28 ± 0.5 °C. After 2 weeks of temporary culture, a full factorial design experiment (MC-LR: 0, 1, 5, 25 μg/L; BC: 0, 100 μg/L) was conducted, including 8 treatment groups. Each treatment group was tested in 2 replicates (30 fish per tank, 16 tanks in total, N = 480), with each tank containing 15 L of aerated water and equipped with a closed flow-through system. The nominal MC-LR concentrations were environmentally relevant and chosen based on Wei et al. [53]. The nominal BC concentration was determined through our preliminary adsorption capacity experiment [54]. To maintain the relatively stable exposure concentrations, 1/3 of the water was replaced every 3 days. MC-LR concentrations were detected using an ELISA kit (Beacon, Saco, ME, USA). The analysis of adsorption capacity is shown in Text S1 and Figure S1, SI. Before sampling, the fish were euthanatized with MS-222 (Sigma-Aldrich, St. Louis, MO, USA). Based on our previous research [34], for each exposure group, the livers from 4 fish were pooled as one replicate, with 3 replicates utilized for antioxidant parameter analysis. Additionally, the livers of 6 fish were collected for qPCR analysis. For histopathological studies, 3 livers were fixed in 10% formalin and 3 livers in 2.5% glutaraldehyde solution. The remaining livers were pooled for MC-LR content detection. This work received approval for research ethics from Hunan University of Arts and Science [Permission number: HUAS-2022-014].

### 4.4. Determination of MC-LR Contents

The MC-LR contents in water and liver samples were determined using ELISA kits (Beacon, Saco, ME, USA), according to previous studies [12,55]. Detailed sample pretreatment and experimental procedures are shown in Text S2, SI.

### 4.5. Histopathological Analysis

After being fixed in formalin for 48 h, the liver samples underwent dehydration through a series of graded ethanol concentrations (70–100%) and were hyalinized in a 1:1 (*v*/*v*) ethanol–xylene mixture. Next, the tissues were embedded in molten paraffin. Paraffin blocks were sectioned (5 μm) using a microtome (Leica, Germany) and stained with hematoxylin and eosin (H&E). All sections were scanned using a digital scanner (Pannoramic MIDI) and observed using CaseViewer (3DHISTECH, Budapest, Hungary). For transmission electron microscopy (TEM) observation, after being pre-fixed in glutaraldehyde overnight, the livers were fixed with osmium tetroxide in phosphate buffer. After washing, the samples were dehydrated through graded ethanol concentrations (50–100%). Next, the samples were embedded, sectioned with an ultramicrotome, stained, and examined using TEM (Hitachi, Japan). A semi-quantitative analysis was utilized to evaluate hepatic histological alterations in the liver of zebrafish, according to Bernet et al. [56], with minor modifications.

### 4.6. Physiological Parameter Analysis

The livers were homogenized in 0.9% saline (1:9, *w*/*v*) and were then centrifuged to obtain the supernatants for further analysis. The physiological parameters, including MDA, SOD, CAT, glutathione peroxidase (GPx), glutathione-s-transferase (GST), and glutathione (GSH), were determined using ELISA kits (Nanjing Jiancheng Bioengineering Institute, Nanjing, China).

### 4.7. qPCR Analysis

TRIzol reagent (Takara, Tokyo, Japan) was utilized to isolate the total RNA in the liver samples. RNA integrity was evaluated using agarose gel electrophoresis. The concentrations of RNA samples were determined using a NanoDrop spectrophotometer (Thermo Scientific, Waltham, MA, USA). Total RNA samples (1 μg) with A260/A280 values ranging from 1.8–2.1 were used for reverse transcription, and the PrimeScript RT reagent Kit (Takara, Japan) with gDNA Eraser was utilized in this step. Next, qPCR was conducted using SYBR Green Kits (Takara, Japan) on a LightCycler^®^ 480 Instrument (Roche, Basel, Switzerland), following the thermal cycling protocols: 40 cycles of 95 °C for 10s, 58 °C for 15s, and 72 °C for 10s. The gene list for the qPCR assay is provided in Table S3, SI, and the primers were designed using Primer Premier 5.0 (Table S4, SI). The *gapdh* gene was used as an endogenous control, and the data were calculated using the 2^−△△Ct^ method [57].

### 4.8. Statistical Analysis

SPSS 25.0 software was used to analyze the data, and all values are presented as mean ± standard deviation (SD). The Kolmogorov–Smirnov test and Levene’s test were used to verify the normality and homogeneity of variance, respectively. One and two-way ANOVA, followed by Tukey’s multiple comparison test, were used to analyze the significant differences between the different treatment groups. Statistical significance was established at *p* < 0.05.

## 5. Conclusions

Our results provide clear evidence that straw-derived BC can significantly decrease MC-LR bioavailability in the liver, thereby alleviating MC-LR-induced hepatic histopathological injury and oxidative damage. Given the widespread occurrence of MC-LR in aquatic environments during cyanobacterial blooms, our findings present a promising approach to mitigating the harmful biological risks associated with MCs. Additionally, our study underscores the importance of exploring different types and forms of BC to further enhance its effectiveness in reducing the detrimental effects of cyanotoxins on fish.

## Figures and Tables

**Table 1:** Semi-quantitative analysis of hepatic H&E-stained sections.

Liver (H&E)	Groups							
	Control	1 μg/L MC-LR	5 μg/L MC-LR	25 μg/L MC-LR	BC	1 + BC	5 + BC	25 + BC
Vacuolization	-	++	++	+++	+	++	++	++
Swollen hepatocytes	-	+	++	+++	-	+	+	-
Pyknotic nuclei	-	+	++	++	-	+	+	+

Note: no damage (-); mild damage (+); moderate damage (++); and severe damage (+++).

**Table 2:** Semi-quantitative analysis of hepatic ultrastructural changes.

Liver (TEM)	Groups							
	Control	1 μg/L MC-LR	5 μg/L MC-LR	25 μg/L MC-LR	BC	1 + BC	5 + BC	25 + BC
Swollen mitochondria	-	-	++	+++	-	-	-	++
Vacuolization	-	+	+	+++	-	-	+	-
Deformed nuclei	-	-	-	+	-	+	-	-
Dilated endoplasmic reticulum	-	-	-	+	-	-	+	-

Note: no damage (-); mild damage (+); moderate damage (++); and severe damage (+++).

## Data Availability

The original contributions presented in this study are included in this article and Supplementary Materials. Further inquiries can be directed to the corresponding authors.

## Supplementary Materials

The following supporting information can be downloaded at: https://www.mdpi.com/article/10.3390/toxins16120549/s1. Figure S1 and Text S1: Adsorption of MC-LR on BC. Text S2: Quantification of MC-LR in the liver and water sample. Table S1: Measured MC-LR levels in the water and liver tissues. Table S2: Transcriptional levels of the Nrf2 signaling pathway genes in the liver. Table S3: Gene list for gene expression assays. Table S4: Sequences of primers used for real-time PCR.

## References

[1] Qin B., Deng J., Shi K., Wang J., Brookes J., Zhou J., Zhang Y., Zhu G., Paerl H., Wu L. (2021). Extreme climate anomalies enhancing cyanobacterial blooms in eutrophic Lake Taihu, China. Water Resour. Res..

[2] Baxa M., Musil M., Kummel M., Hanzlík P., Tesařová B., Pechar L. (2021). Dissolved oxygen deficits in a shallow eutrophic aquatic ecosystem (fishpond)-Sediment oxygen demand and water column respiration alternately drive the oxygen regime. Sci. Total Environ..

[3] You L., Tong X., Te S.H., Tran N.H., bte Sukarji N.H., He Y., Gin K.Y.H. (2022). Multi-class secondary metabolites in cyanobacterial blooms from a tropical water body: Distribution patterns and real-time prediction. Water Res..

[4] Baliu-Rodriguez D., Peraino N.J., Premathilaka S.H., Birbeck J.A., Baliu-Rodriguez T., Westrick J.A., Isailovic D. (2022). Identification of novel microcystins using high-resolution MS and MS^n^ with python code. Environ. Sci. Technol..

[5] Xie G., Hu X., Du Y., Jin Q., Liu Y., Tang C., Hu X., Li G., Chen Z., Zhou D. (2021). Light-driven breakdown of microcystin-LR in water: A critical review. Chem. Eng. J..

[6] Wang Q., Niu Y., Xie P., Chen J., Ma Z., Tao M., Qi M., Wu L., Guo L. (2010). Factors affecting temporal and spatial variations of microcystins in Gonghu Bay of Lake Taihu, with potential risk of microcystin contamination to human health. Sci. World J..

[7] Saoudi A., Brient L., Boucetta S., Ouzrout R., Bormans M., Bensouilah M. (2017). Management of toxic cyanobacteria for drinking water production of Ain Zada Dam. Environ. Monit. Assess..

[8] Greer B., Meneely J.P., Elliott C.T. (2018). Uptake and accumulation of Microcystin-LR based on exposure through drinking water: An animal model assessing the human health risk. Sci. Rep..

[9] Schaefer A.M., Yrastorza L., Stockley N., Harvey K., Harris N., Grady R., Sullivan J., McFarland M., Reif J.S. (2020). Exposure to microcystin among coastal residents during a cyanobacteria bloom in Florida. Harmful Algae.

[10] Malbrouck C., Kestemont P. (2006). Effects of microcystins on fish. Environ. Toxicol. Chem..

[11] Mohamed Z.A., Carmichael W.W., Hussein A.A. (2003). Estimation of microcystins in the freshwater fish *Oreochromis niloticus* in an Egyptian fish farm containing a *Microcystis* bloom. Environ. Toxicol..

[12] Flores N.M., Miller T.R., Stockwell J.D. (2018). A global analysis of the relationship between concentrations of microcystins in water and fish. Front. Mar. Sci..

[13] Le Manach S., Sotton B., Huet H., Duval C., Paris A., Marie A., Yépremian C., Catherine A., Mathéron L., Vinh J. (2018). Physiological effects caused by microcystin-producing and non-microcystin producing *Microcystis aeruginosa* on medaka fish: A proteomic and metabolomic study on liver. Environ. Pollut..

[14] Shahmohamadloo R.S., Ortiz Almirall X., Simmons D.B., Lumsden J.S., Bhavsar S.P., Watson-Leung T., Eyken A.V., Hankins G., Hubbs K., Konopelko P. (2021). Cyanotoxins within and outside of Microcystis aeruginosa cause adverse effects in rainbow trout (*Oncorhynchus mykiss*). Environ. Sci. Technol..

[15] Shahmohamadloo R.S., Almirall X.O., Simmons D.B., Poirier D.G., Bhavsar S.P., Sibley P.K. (2022). Fish tissue accumulation and proteomic response to microcystins is species-dependent. Chemosphere.

[16] Qiao Q., Djediat C., Huet H., Duval C., Le Manach S., Bernard C., Edery M., Marie B. (2019). Subcellular localization of microcystin in the liver and the gonads of medaka fish acutely exposed to microcystin-LR. Toxicon.

[17] Fischer W.J., Hitzfeld B.C., Tencalla F., Eriksson J.E., Mikhailov A., Dietrich D.R. (2000). Microcystin-LR toxicodynamics, induced pathology, and immunohistochemical localization in livers of blue-green algae exposed rainbow trout (*Oncorhynchus mykiss*). Toxicol. Sci..

[18] Shi L., Du X., Liu H., Chen X., Ma Y., Wang R., Tian Z., Zhang S., Guo H., Zhang H. (2021). Update on the adverse effects of microcystins on the liver. Environ. Res..

[19] Zhang H., Zhao X., Li Y., Xie P. (2023). A meta-analysis on the toxicity of microcystin-LR to fish and mammals. Environ. Pollut..

[20] He Y., Ouyang K., Yang H., Wang L., Wang X., Li D., Li L. (2024). The impact of ammonia and microcystin-LR on neurobehavior and glutamate/gamma-aminobutyric acid balance in female zebrafish (*Danio rerio*): ROS and inflammation as key pathways. Sci. Total Environ..

[21] Wan X., Cheng C., Gu Y., Shu X., Xie L., Zhao Y. (2021). Acute and chronic toxicity of microcystin-LR and phenanthrene alone or in combination to the cladoceran (*Daphnia magna*). Ecotoxicol. Environ. Saf..

[22] Xiao Y., Hu L., Duan J., Che H., Wang W., Yuan Y., Xu J., Chen D., Zhao S. (2024). Polystyrene microplastics enhance microcystin-LR-induced cardiovascular toxicity and oxidative stress in zebrafish embryos. Environ. Pollut..

[23] Tomczyk A., Sokołowska Z., Boguta P. (2020). Biochar physicochemical properties: Pyrolysis temperature and feedstock kind effects. Rev. Environ. Sci. Bio/Technol..

[24] Palansooriya K.N., Yang Y., Tsang Y.F., Sarkar B., Hou D., Cao X., Meers E., Rinklebe J., Kim K.H., Ok Y.S. (2020). Occurrence of contaminants in drinking water sources and the potential of biochar for water quality improvement: A review. Crit. Rev. Environ. Sci. Technol..

[25] Ambaye T.G., Vaccari M., van Hullebusch E.D., Amrane A., Rtimi S.J.I.J.O.E.S. (2021). Mechanisms and adsorption capacities of biochar for the removal of organic and inorganic pollutants from industrial wastewater. Int. J. Environ. Sci. Technol..

[26] Li J., Cao L., Yuan Y., Wang R., Wen Y., Man J. (2018). Comparative study for microcystin-LR sorption onto biochars produced from various plant-and animal-wastes at different pyrolysis temperatures: Influencing mechanisms of biochar properties. Bioresource Technol..

[27] Wei L., Lu J. (2021). Adsorption of microcystin-LR by rice straw biochars with different pyrolysis temperatures. Environ. Technol. Innov..

[28] Horzmann K.A., Freeman J.L. (2018). Making waves: New developments in toxicology with the zebrafish. Toxicol. Sci..

[29] Cha J.S., Park S.H., Jung S.C., Ryu C., Jeon J.K., Shin M.C., Park Y.K. (2016). Production and utilization of biochar: A review. J. Ind. Eng. Chem..

[30] Liu B.L., Fu M.M., Xiang L., Feng N.X., Zhao H.M., Li Y.W., Cai Q.Y., Li H., Mo C.H., Wong M.H. (2021). Adsorption of microcystin contaminants by biochars derived from contrasting pyrolytic conditions: Characteristics, affecting factors, and mechanisms. Sci. Total Environ..

[31] Zeng S., Kan E. (2021). Adsorption and regeneration on iron-activated biochar for removal of microcystin-LR. Chemosphere.

[32] Minarik T.A., Vick J.A., Schultz M.M., Bartell S.E., Martinovic-Weigelt D., Rearick D.C., Schoenfuss H.L. (2014). On-site exposure to treated wastewater effluent has subtle effects on male fathead minnows and pronounced effects on carp. J. Am. Water Resour. Assoc..

[33] Atencio L., Moreno I., Prieto A.I., Moyano R., Molina A.M., Cameán A.M. (2008). Acute effects of microcystins MC-LR and MC-RR on acid and alkaline phosphatase activities and pathological changes in intraperitoneally exposed tilapia fish (*Oreochromis* sp.). Toxicol. Pathol..

[34] Lin W., Hou J., Guo H., Li L., Wang L., Zhang D., Li D., Tang R. (2018). The synergistic effects of waterborne microcystin-LR and nitrite on hepatic pathological damage, lipid peroxidation and antioxidant responses of male zebrafish. Environ. Pollut..

[35] Wolf J.C., Wheeler J.R. (2018). A critical review of histopathological findings associated with endocrine and non-endocrine hepatic toxicity in fish models. Aquat. Toxicol..

[36] Martemucci G., Costagliola C., Mariano M., D’andrea L., Napolitano P., D’Alessandro A.G. (2022). Free radical properties, source and targets, antioxidant consumption and health. Oxygen.

[37] Ott M., Gogvadze V., Orrenius S., Zhivotovsky B. (2007). Mitochondria, oxidative stress and cell death. Apoptosis.

[38] Hou J., Li L., Xue T., Long M., Su Y., Wu N. (2015). Hepatic positive and negative antioxidant responses in zebrafish after intraperitoneal administration of toxic microcystin-LR. Chemosphere.

[39] Cao N., Zong X., Guo X., Chen X., Nie D., Huang L., Li L., Ma Y., Wang C., Pang S. (2024). The adsorption effects of biochar on carbofuran in water and the mixture toxicity of biochar-carbofuran in rats. Chemosphere.

[40] Spahis S., Delvin E., Borys J.M., Levy E. (2017). Oxidative stress as a critical factor in nonalcoholic fatty liver disease pathogenesis. Antioxid. Redox Sign..

[41] Shi Y., Jiang J., Shan Z., Bu Y., Deng Z., Cheng Y. (2015). Oxidative stress and histopathological alterations in liver of *Cyprinus carpio* L. induced by intraperitoneal injection of microcystin-LR. Ecotoxicology.

[42] Reda R.M., El Gaafary N.M., Rashwan A.A., Mahsoub F., El-Gazzar N. (2022). Evaluation of olive stone biochar as valuable and inexpensive agro-waste adsorbent for the adsorption and removal of inorganic mercury from Nile tilapia aquaculture systems. Aquac. Res..

[43] Hoseinifar S.H., Yousefi S., Van Doan H., Ashouri G., Gioacchini G., Maradonna F., Carnevali O. (2020). Oxidative stress and antioxidant defense in fish: The implications of probiotic, prebiotic, and synbiotics. Rev. Fish. Sci. Aquac..

[44] Abele D., Vazquez-Medina J.P., Zenteno-Savin T. (2011). Oxidative Stress in Aquatic Ecosystems.

[45] Ming J., Ye J., Zhang Y., Yang X., Shao X., Qiang J., Xu P. (2019). Dietary optimal reduced glutathione improves innate immunity, oxidative stress resistance and detoxification function of grass carp (*Ctenopharyngodon idella*) against microcystin-LR. Aquaculture.

[46] Wu P., Zhang L., Jiang W., Liu Y., Jiang J., Kuang S., Li S., Tang L., Tang W., Zhou X. (2022). Dietary vitamin A improved the flesh quality of grass carp (*Ctenopharyngodon idella*) in relation to the enhanced antioxidant capacity through Nrf2/Keap 1a signaling pathway. Antioxidants.

[47] Yang J., Zhang Z., Du X., Wang Y., Meng R., Ge K., Wu C., Liang X., Zhang H., Guo H. (2024). The effect and mechanism of combined exposure of MC-LR and NaNO_2_ on liver lipid metabolism. Environ. Res..

[48] Liu M.J., Guo H.Y., Zhu K.C., Liu B.S., Liu B., Guo L., Zhang N., Yang J.Y., Jiang S.G., Zhang D.C. (2021). Effects of acute ammonia exposure and recovery on the antioxidant response and expression of genes in the Nrf2-Keap1 signaling pathway in the juvenile golden pompano (*Trachinotus ovatus*). Aquat. Toxicol..

[49] Bian L., Nguyen V.T., Tamaoki J., Endo Y., Dong G., Sato A., Kobayashi M. (2023). Genetic hyperactivation of Nrf2 causes larval lethality in Keap1a and Keap1b-double-knockout zebrafish. Redox Biol..

[50] Wu J.L., Liu W.X., Wen C.G., Qian G.M., Hu B.Q., Jian S.Q., Yang G., Dong J. (2020). Effect of microcystin on the expression of Nrf2 and its downstream antioxidant genes from *Cristaria plicata*. Aquat. Toxicol..

[51] Zhang H., Xie P. (2023). The mechanisms of microcystin-LR-induced genotoxicity and neurotoxicity in fish and mammals: Bibliometric analysis and meta-analysis. Sci. Total Environ..

[52] Kumari T., Phogat D., Phogat J., Shukla V. (2024). Biochar & fly ash amendments lower mortality and increase antioxidant activity in chlorpyrifos-exposed earthworms. Appl. Biol. Chem..

[53] Wei H., Jia Y., Wang Z. (2022). Microcystin pollution in lakes and reservoirs: A nationwide meta-analysis and assessment in China. Environ. Pollut..

[54] Lin W., Guo H., Yang L., Kuang Y., Li D., Yang P., Li L. (2022). Alleviation of microcystin-LR-induced hepatic lipidosis and apoptosis in zebrafish by use of rice straw-derived biochar. Ecotoxicol. Environ. Saf..

[55] Wu Q., Yan W., Cheng H., Liu C., Hung T.C., Guo X., Li G. (2017). Parental transfer of microcystin-LR induced transgenerational effects of developmental neurotoxicity in zebrafish offspring. Environ. Pollut..

[56] Bernet D., Schmidt H., Meier W., Burkhardt-Holm P., Wahli T. (1999). Histopathology in fish: Proposal for a protocol to assess aquatic pollution. J. Fish Dis..

[57] Livak K.J., Schmittgen T.D. (2001). Analysis of relative gene expression data using real-time quantitative PCR and the 2^− ΔΔCT^ method. Methods.

