# Supplementary material for: Rice Straw-Derived Biochar Mitigates Microcystin-LR-Induced Hepatic Histopathological Injury and Oxidative Damage in Male Zebrafish via the Nrf2 Signaling Pathway
Source: Toxins (Basel). 2024 Dec 18;16(12):549. doi: 10.3390/toxins16120549 (PMC11679041; doi:10.3390/toxins16120549)
Supplement: Supplementary file 1 [file toxins-16-00549-s001.zip › toxins-3349525-supplementary.pdf]

Supporting Information

Rice straw-derived biochar mitigates microcystin-LR-induced hepatic histopathological injury and oxidative damage in male zebrafish via the Nrf2 signaling pathway

Wang Lin [1,2,3], Fen Hu [1], Wansheng Zou [1,3], Suqin Wang [1,3], Pengling Shi [1,3], Li Li [4], Jifeng Yang [5,*], Pinhong Yang [1,3,*]

[1] College of Life and Environmental Sciences, Hunan University of Arts and Science, Changde 415000, China

[2] Institute for Ecological Research and Pollution Control of Plateau Lakes, School of Ecology and Environmental Science, Yunnan University, Kunming 650500, China

[3] Hunan Provincial Key Laboratory for Molecular Immunity Technology of Aquatic Animal Diseases, Changde 415000, China

[4] College of Chemistry and Materials Engineering, Hunan University of Arts and Science, Changde 415000, China

[5] College of Fisheries, Huazhong Agricultural University, Wuhan 430070, China

***Corresponding Author:**

Hunan University of Arts and Science

No.3150 Dongting Avenue, Changde 415000, P.R. China.

E-mail address: ji-feng.yang@huas.edu.cn (J.Y.); yph0102@huas.edu.cn (P.Y.)

Number of pages: 8, number of Texts: 2, number of Figures: 1, number of Tables: 4.

This document provides supporting information about the adsorption analysis, quantification of MC-LR, and qPCR analysis.

Contents

Text S1 & Fig. S1: Adsorption of MC-LR on BC.

Text S2: Quantification of MC-LR in the liver and water sample.

Table S1. Measured MC-LR levels in the water and liver tissues.

Table S2: Transcriptional levels of the Nrf2 signaling pathway genes in the liver.

Table S3: Gene list for gene expression assays.

Table S4: Sequences of primers used for real-time PCR.

Text S1 & Fig. S1. Adsorption of MC-LR on BC.

To evaluate the MC-LR adsorption capacity on BC for MC-LR, a solution containing 25 μg/L MC-LR and 100 μg/L BC was prepared and shaken at room temperature for 24 h. Next, 5 mL samples were taken from the mixture every 4 h, and then centrifuged at 3000 rpm for 10 min. The supernatant was used for MC-LR analysis, which indicates the amount of MC-LR not adsorbed by BC.

As shown in Fig. S1, in the MC-LR-only group, the MC-LR concentrations decreased from 24.02 ± 0.77 μg/L to 20.66 ± 1.55 μg/L, representing a 13.99 % reduction after 24 h. In contrast, in the mixture of MC-LR and BC, the MC-LR concentrations decreased from 23.95 ± 0.91 μg/L to 17.74 ± 1.11 μg/L, with a 25.93 % reduction after 24 h. And there is significant difference between the MC-LR + BC group and MC-LR group ($P < 0.05$).

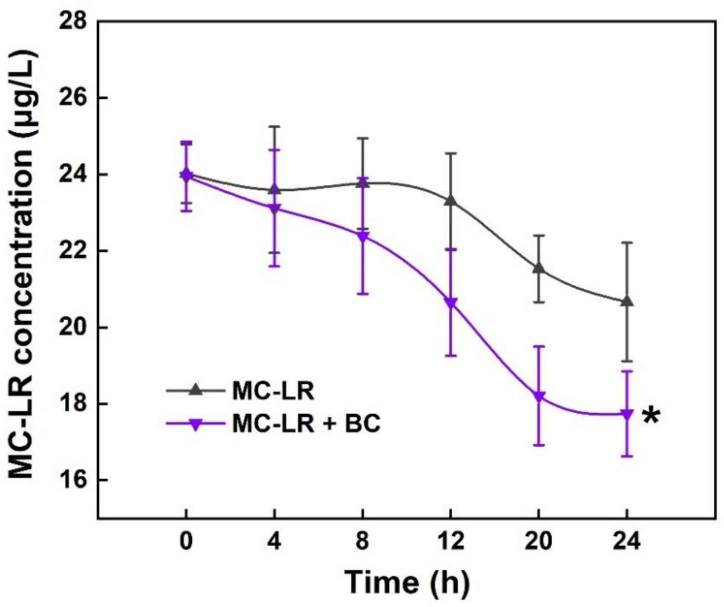

Figure S1. Adsorption analysis of BC (100 μg/L) on MC-LR (25 μg/L) in water. Asterisks (*) indicate significant differences between the MC-LR + BC group and the MC-LR group (* $P < 0.05$). The values are presented as mean ± SD (n = 3).

Text S2. Quantification of MC-LR in the liver and water sample.

Liver samples were extracted using a solvent mixture of methanol, butanol, and water in a ratio of 20: 5: 75 (v/v/v), and the extracts were passed through a C18 cartridge. The cartridge was washed with 20 mL of distilled water, and samples were sequentially eluted with 20 mL of 20% methanol followed by 20 mL of 100% methanol. The final elution fraction was dried and re-dissolved in water for further analysis. MC-LR exposure water was filtered through Whatman GF/C filters (1.4 μm), transferred to new centrifuge tubes, and stored at -20 °C for further analysis.

MC-LR quantification was performed using commercial ELISA kits (Beacon, USA). Specifically, the microcystin-HRP enzyme conjugate solution (50 μL per well) was added to each well. Next, the negative control, samples, and standard solutions were separately added to the wells, followed by the rabbit anti-microcystin antibody. The plate was covered with parafilm and incubated on a shaking table in the dark for 30 min. After incubation, the plate was washed five times with 1× cleansing solution, followed by the addition of 100 μL substrate solution to each well. After reacting at 37 °C for 30 min, 100 μL stop solution was added to all wells. Finally, the absorbance was measured at 450 nm using a microplate reader (BioTek, USA). The detection limit for MC-LR was 0.1 μg/L.

Table S1. Measured MC-LR levels in the water and liver tissues.

Group	Water (μg/L)		Liver (ng/g WW)
	Before	After	
Control	< MDL	< MDL	< MDL
1 μg/L MC-LR	0.81 ± 0.04	1.01 ± 0.02	1.70 ± 0.09
5 μg/L MC-LR	3.94 ± 0.29	5.17 ± 0.38	2.14 ± 0.06
25 μg/L MC-LR	20.33 ± 0.77	25.10 ± 0.69	4.73 ± 0.11
100 μg/L BC	< MDL	< MDL	< MDL
1 μg/L MC-LR + BC	0.61 ± 0.07**	0.99 ± 0.03	1.51 ± 0.04
5 μg/L MC-LR + BC	3.06 ± 0.20**	4.91 ± 0.29	1.92 ± 0.05
25 μg/L MC-LR + BC	14.80 ± 0.82**	25.01 ± 1.23	2.07 ± 0.09**

Note: Asterisks indicate significant differences (* $P < 0.05$, ** $P < 0.01$) between the MC-LR + BC groups and the corresponding MC-LR groups by using an independent t-test. WW: Wet weight. MDL: Minimum detection limit (0.1 μg/L). The values are presented as mean ± SD (n = 3).

Table S2. Transcriptional levels of the Nrf2 signaling pathway genes in the liver.

Gene	Groups							
		MC-LR exposure groups				MC-LR + BC exposure groups		
	Control	1 μg/L	5 μg/L	25 μg/L	BC	1 μg/L + BC	5 μg/L + BC	25 μg/L + BC
cat	1.08 ± 0.45	1.27 ± 0.34	0.89 ± 0.32	0.40 ± 0.21*	1.09 ± 0.30	1.21 ± 0.39	1.02 ± 0.29	0.80 ± 0.27#
sod1	1.05 ± 0.34	1.07 ± 0.51	0.80 ± 0.35	0.41 ± 0.15*	1.18 ± 0.56	1.04 ± 0.53	0.97 ± 0.31	0.79 ± 0.24##
gpx1a	1.04 ± 0.33	1.26 ± 0.41	1.15 ± 0.29	0.72 ± 0.30	1.22 ± 0.41	1.03 ± 0.55	1.27 ± 0.42	1.09 ± 0.40
gstr	1.09 ± 0.45	1.50 ± 0.46	1.29 ± 0.61	0.70 ± 0.34	0.93 ± 0.32	1.20 ± 0.61	1.86 ± 0.62	1.33 ± 0.30#
keap1a	1.06 ± 0.40	1.12 ± 0.32	0.90 ± 0.12	0.39 ± 0.12*	0.92 ± 0.36	0.90 ± 0.40	0.89 ± 0.40	0.71 ± 0.22#
keap1b	1.05 ± 0.37	1.34 ± 0.40	1.06 ± 0.55	0.55 ± 0.11	0.88 ± 0.24	1.08 ± 0.24	0.79 ± 0.41	0.79 ± 0.37
nrf2a	1.07 ± 0.42	1.17 ± 0.71	1.80 ± 0.88	2.49 ± 0.72**	1.27 ± 0.19	1.23 ± 0.48	1.57 ± 0.43	1.28 ± 0.37##
nrf2b	1.10 ± 0.48	1.29 ± 0.55	1.54 ± 0.57	2.09 ± 0.79*	1.07 ± 0.41	0.94 ± 0.47	1.23 ± 0.26	1.32 ± 0.49
nqo1	1.06 ± 0.40	1.44 ± 0.38	1.27 ± 0.52	1.42 ± 0.59	1.12 ± 0.23	1.32 ± 0.32	1.43 ± 0.80	1.10 ± 0.22
gclm	1.08 ± 0.39	1.13 ± 0.39	1.37 ± 0.55	1.40 ± 0.35	1.32 ± 0.28	0.96 ± 0.40	1.38 ± 0.30	0.98 ± 0.42
gclc	1.07 ± 0.37	1.28 ± 0.44	1.29 ± 0.32	1.94 ± 0.45**	0.95 ± 0.23	1.02 ± 0.30	1.18 ± 0.43	1.24 ± 0.36#
hmox1a	1.03 ± 0.30	1.21 ± 0.36	1.41 ± 0.44	1.89 ± 0.48**	1.01 ± 0.34	1.03 ± 0.42	1.17 ± 0.22	1.33 ± 0.39

Notes: Transcriptional levels of target genes are expressed as the fold change relative to the control. Asterisks (*) and (**) indicate significant differences at $P < 0.05$ and $P < 0.01$ between the treatments and control. Hashes (#) and (##) indicate significant differences at $P < 0.05$ and $P < 0.01$ between the MC-LR + BC groups and the corresponding MC-LR groups, respectively. The values are presented as mean ± SD (n = 6).

Table S3. Gene list for gene expression assays.

Abbreviations	Gene name
cat	catalase
sod1	superoxide dismutase 1
gpx1a	glutathione peroxidase 1a
gstr	glutathione S-transferase rho
keap1a	kelch-like ECH-associated protein 1a
keap1b	kelch-like ECH-associated protein 1b
nrf2a	nfe2 like bZIP transcription factor 2a
nrf2b	nfe2 like bZIP transcription factor 2b
nqo1	NAD(P)H dehydrogenase, quinone 1
gclm	glutamate-cysteine ligase, modifier subunit
gclc	glutamate-cysteine ligase, catalytic subunit
hmox1a	heme oxygenase 1a
gapdh	glyceraldehyde-3-phosphate dehydrogenase

Table S4. Sequences of primers used for real-time PCR.

Target gene	Accession No.	Primer sequences (from 5' to 3')	Product length (bp)
cat	BC051626	F: CAAGGTCTGGTCCCATAAA	226
		R: GACTGGTAGTTGGAGGTAA	
sod1	BC055516	F: GTCCGCACTTCAACCCTCA	217
		R: TCCTCATTGCCACCCTTCC	
gpx1a	BC083461	F: GGCACAACAGTCAGGGATT	239
		R: AGGAACGCAAACAGAGGG	
gstr	NM_001045060	F: CCAACATTCAAGCACGGAG	198
		R: CACAAGCCAATCATAAAAGG	
keap1a	NM_182864	F: TGATGGACAAACCCAACTCA	167
		R: CACTGGACAGGAAACCACCT	
keap1b	NM_001113477	F: ACGAGTGGAGGAGCATAGCG	247
		R: AACGTGTTCCCATCATAGCC	
nrf2a	NM_182889	F: TGGCCCTGAAGAATTTAACG	138
		R: CCCGGTGAGAAGCTCTGTAG	
nrf2b	HQ661166	F: CCTGCCCAACAGACTCTCTC	136
		R: CGTCTTTGTCCGACTGTTCA	
nqo1	NM_205542	F: CTGGGTGGTGTGTTTGAAGAA	137
		R: GCTGTGGTAATGCCGTAGG	
gclm	XM_009303802	F: TCGCTCCTCCTTCTCTTCC	109
		R: CCGATGGCAGCAATCTTCT	
gclc	NM_199277	F: CGGATGGAGAGTGGAGTTCA	170
		R: TTCGCTTCTGGGCTACCTT	
hmox1a	NM_001127516	F: GGAAGAGCTGGACAGAAACG	107
		R: CGAAGAAGTGCTCCAAGTCC	
gapdh	BC095386	F: CTGGTGACCCGTGCTGCTT	150
		R: TTTGCCGCCTTCTGCCTTA	

Note: F-Forward; R-Reverse
